# PDZK1 is correlated with DCE-MRI perfusion parameters in high-grade glioma

**DOI:** 10.1016/j.clinsp.2024.100367

**Published:** 2024-04-30

**Authors:** Yi Zhang, Feng Wang, YongLi Huang

**Affiliations:** Department of Radiology, The First People's Hospital of Shuangliu District, (West China Airport Hospital of Sichuan University), Chengdu City, Sichuan Province, China

**Keywords:** Pdzk1, High-grade glioma, Prognosis, DCE-MRI, Perfusion parameter

## Abstract

**Objective:**

This study investigated the relationship between PDZK1 expression and Dynamic Contrast-Enhanced MRI (DCE-MRI) perfusion parameters in High-Grade Glioma (HGG).

**Methods:**

Preoperative DCE-MRI scanning was performed on 80 patients with HGG to obtain DCE perfusion transfer coefficient (K^trans^), vascular plasma volume fraction (v_p_), extracellular volume fraction (v_e_), and reverse transfer constant (k_ep_). PDZK1 in HGG patients was detected, and its correlation with DCE-MRI perfusion parameters was assessed by the Pearson method. An analysis of Cox regression was performed to determine the risk factors affecting survival, while Kaplan-Meier and log-rank tests to evaluate PDZK1′s prognostic significance, and ROC curve analysis to assess its diagnostic value.

**Results:**

PDZK1 was upregulated in HGG patients and predicted poor overall survival and progression-free survival. Moreover, PDZK1 expression distinguished grade III from grade IV HGG. PDZK1 expression was positively correlated with K^trans 90^, and v_e_90_, and negatively correlated with k_ep_max_, and k_ep_90_.

**Conclusion:**

PDZK1 is upregulated in HGG, predicts poor survival, and differentiates tumor grading in HGG patients. PDZK1 expression is correlated with DCE-MRI perfusion parameters.

## Highlights

• PDZK1 is up-regulated in cancer tissues of HGG patients.
• PDZK1 expression was significantly positively correlated with k^trans^_90_, v_e_90_.
• PDZK1 expression was negatively correlated with k_ep_max_, k_ep_90_.
• Patients with high PDZK1 expression had poor OS and PFS.

## Introduction

High-Grade Glioma (HGG) including Glioblastoma (GBM) is the most aggressive brain tumor. Regardless of a combination of treatments, GBM patients have a short survival period,^1^ with 8 % surviving 2.5 years or more.^2^ WHO glioma classification has been considered the most reliable prognostic factor, but this view has been challenged by HGG genetic and imaging studies.^3^, ^4^, ^5^ Perfusion parameters are recognized as an independent predictor of survival.^6^^,^^7^ Perfusion parameters are associated with tumor vascular distribution and vascular properties, which are closely related to tumor progression and patients’ survival.^8^^,^^9^ Thus, in addition to glioma stratification, perfusion is considered to predict survival and ultimately help select treatment.

Dynamic Susceptibility Contrast (DSC) and Dynamic Contrast-Enhanced (DCE) perfusion are common in the field of MRI. There are considerable differences between the two techniques. First, DSC perfusion is determined by changes in T2 signals when contrast agents are present, and by changes in T1 signals when enhanced contrast is present for DCE. Second, DSC mainly measures cerebral blood volume and quantifies the number of blood vessels, while DCE perfusion focuses on vascular quality and quantifies vascular permeability. Increased vascular permeability is the main feature of neovascularization, which is reflected in the increase of perfusion transfer coefficient (K^trans^). DCE perfusion can also calculate extracellular volume fraction (v_e_), reverse transfer constant (k_ep_), and vascular plasma volume fraction (v_p_). Studies have shown that DCE perfusion parameters possess diagnostic values in glioma tumor grading.^10^^,^^11^

As a PDZ protein, PDZK1 containing four PDZ domains has significant actions on tumor growth, metastasis, and drug resistance.^12^ PDZK1 expression and function have been confirmed for breast cancer^13^ and cervical cancer.^14^ This study was to investigate the relationship between PDZK1 expression and DCE-MRI perfusion parameters and its prognostic value in HGG patients.

## Materials and methods

### Patient population

This study was an observational clinical study following the Strengthening the Reporting of Observational Studies in Epidemiology (STROBE) guidelines. This study was approved by the Ethics Committee of The First People's Hospital of Shuangliu District (n° 201906SC29). All relevant information has been detailed to the patient and informed consent has been obtained. Eighty patients with primary HGG (anaplastic astrocytoma or GBM) were included according to conventional or spectroscopic MRI findings. Exclusion criteria (1) Any previous brain tumor; (2) Other tumor histology; (3) No informed consent.

DCE-MRI was performed before surgery to confirm tumor histology. Variables such as age, gender, and treatment were recorded during the study. All patients were treated with surgery, and most also experienced adjuvant therapy, such as chemotherapy, radiation, or combination therapy. During follow-up regularly. MRI was performed postoperatively, at 3-weeks, 2, 3, 6, and 12-months after chemotherapy began, and was followed regularly until clinical deterioration, such as neurological deterioration, radiological progression, or death. Response Assessment criteria defined radiological progression as the duration of initial chemotherapy.^15^

### MRI

A 1.5T scanner (MAGNETOM Avanto, Siemens Healthcare, Germany) and 12-channel array head coils were utilized in MRI examinations. For axial FLAIR images, TR/TE was 99/94 ms, TI was 2500 ms, slice thickness was 4 mm, intersection gap was 10 %, and field of view was 220 × 220 mm. For distortion-corrected T1-weighted image, TR/TE was 275/2.5 ms, intersection gap was 10 %, slice thickness was 4 mm, and field of view was 230 × 230 mm. The 3D fast Low Angle Shooting (FLASH) sequence was used to optimize the temporal and spatial resolution, with TR/TE at 4/1.4 ms, flip angle at 15°, temporal resolution for 6 s, phase resolution at 100 %, base resolution at 128, slice resolution at 100 %, GRAPPA factor as 2, slice thickness of 4 mm, field of view of 220 × 220 mm, for 5 min totally. Signal strength was converted into gadolinium concentration in T1 mapping. The T1 plot was calculated by pre-comparing multiple flip angles (6°, 12°, and 15° for 1 min). Other acquisition parameters were similar. Given that the tumor is always at the center of the imaging volume, nominal and effective flip angles are considered with negligible differences. Gadobinol (0.1 mmoL/kg)at 4 mL/s was administered, and saline irrigation was conducted.

### Image analysis

The entire tumor volume was manually mapped offline in T1-enhanced images. To correct motion and register rigid-body models in pre-contrast MRI images, Olea SphereTM software version 2.3 was used, converting signal strength to gadolinium concentration. Image alignment was calibrated with visual validation and adjustments. V_e_, K^trans^, and v_p_ were provided by the software based on the improved Tofts-Kermode model.^16^ K_ep_ = K^trans^/v_e_. The standard singular value decomposition method was used for deconvolution calculation. For the best-fitted Arterial Input Function (AIF) curve, either the right or left internal carotid artery C4 segment was selected. Not all patients had good basilar or middle cerebral arteries, so the internal carotid artery was imaged. AIF is model-based and dose-by-dose using a double exponential function.^17^ The calculated measurement unit of DCE-MRI parameters was K^trans^ and k_ep_ per minute, and v_p_ and v_e_ dimensionless.^18^ In contrast-enhanced T1-weighted images (without any large blood vessels), the area of interest was drawn around the tumor. If no contrast enhancement was observed, it was drawn around the high-intensity tumor area on the FLAIR image. Follow-up data were evaluated blind. Post-processing was repeated for each section containing tumor tissue, and all ROI parameters were exported. The voxels successfully fitted were further analyzed, the criteria being that all perfusion parameters were non-negative, 0 < K^trans^ < 4, 0 < v_e_ < 1.

### RT-qPCR

Cancerous and para-cancerous tissues of HGG patients were obtained during the operation. Total RNA was isolated using TRIzol Reagent (Life Technologies, CA, USA). Total RNA (1 μg) was reverse transcribed using a QuantiTect Reverse Transcription kit (Qiagen, CA, USA). SYBR Green Master Mix (Applied Biosystems, USA) was utilized for RT-qPCR performed on Applied Biosystems Prism 7900HT Sequence Detection System (PE Applied Biosystems). PDZK1 (F: 5′-AGGATCAATGGTGTCTTTGTCG-3′ and R: 5′-TCCAGCTCTTTCAAATCCACC-3′); GADPH (F: 5′-ACAGTCAGCCGCATCTTCTT-3 'and R: 5′-AAATGAGCCCCAGCCTTCTC-3′).

### Western blot

Total protein was extracted by RIPA lysis buffer (Beyotime, China). For measuring protein concentration, a BCA kit (Beyotime) was purchased. The total protein was isolated using 12 % SDS-PAGE, transferred to a PVDF membrane (Millipore), blocked with 5 % skim milk, incubated with primary antibody anti-PDZK1 (ab92491, 1:1000, Abcam, UK) and GADPH (ab45171, 1:1000, Abcam, UK) at 4 °C overnight, and re-detected with a secondary antibody (Cell Signaling Technology, USA) for 2 h. Signals were enhanced by adding an ECL substrate (Millipore). Protein gray values were analyzed by Image J software.

### Statistical analysis

Overall Survival (OS) refers to days from the date of DCE-MRI examination to the date of death or last available follow-up, and Progression-Free Survival (PFS) refers to days from clinical and MRI validation to progression, or if there is no progression or death, to the last follow-up date. Patients were stratified using 1-year PFS and OS as dividing lines.

Kolmogorov-Smirnov was utilized to test the normality of the data. Student *t*-test was used to statistically test the differences. Pearson correlation analysis was utilized to evaluate the correlation between PDZK1 and DCE-MRI perfusion parameters. An analysis of Cox regression was performed to determine the risk factors affecting survival, while Kaplan-Meier and log-rank tests to evaluate PDZK1′s prognostic significance, and ROC curve analysis to assess its diagnostic value. Continuous variables were represented as mean ± standard deviation and discrete variables were represented as median with range. The maximum value (__max_), 90th percentile (__90_), skewness (__skew_), and kurtosis (__kurt_) were calculated. Statistical analysis was performed using SPSS version 22, with *p* < 0.05 indicating statistical significance.

## Results

### Patient information

Among 80 patients with HGG, 48 were male and 32 were female; 56 tumors were grade IV (GBM) and 24 were grade III (anaplastic astrocytoma). The median age was 55 years (22‒76 years). Patients were followed for a median of 420 days (21‒1830 days). During follow-up, 64 patients developed tumor progression and 52 patients died. In addition, 14 patients received radiation only, 3 patients received chemotherapy, and 63 patients received combined therapy.

### Descriptive statistics of perfusion parameters

After removing unsuitable voxels, 2090 (median, range 90‒17,590) voxels were analyzed. Table 1 summarizes the perfusion parameters. The authors found significant differences in K^trans^_90_, v_e_90_, k_ep_max_, and k_ep_90_ between grade III and IV HGG.

**Table 1:** Summary of patient perfusion parameters.

		Glioma grade		
Perfusion parameters	High-grade glioma (*n* = 80)	Grade III (*n* = 24)	Grade IV (*n* = 56)	p
Ktrans max	0.48 ± 0.22	0.43 ± 0.21	0.50 ± 0.24	0.197
Ktrans 90	0.12 ± 0.07	0.06 ± 0.03	0.15 ± 0.07	<0.001
Ktrans skew	3.85 ± 1.75	4.15 ± 2.04	3.72 ± 1.76	0.343
Ktrans kurt	46.47 ± 20.64	55.64 ± 32.50	42.52 ± 21.88	0.08
vp_max	0.35 ± 0.13	0.31 ± 0.12	0.37 ± 0.17	0.078
vp_90	0.12 ± 0.06	0.11 ± 0.05	0.13 ± 0.07	0.154
vp_skew	2.57 ± 1.28	2.21 ± 1.15	2.72 ± 1.46	0.1
vp_kurt	12.84 ± 7.22	11.07 ± 5.26	13.60 ± 6.71	0.076
ve_max	0.88 ± 0.32	0.78 ± 0.42	0.92 ± 0.55	0.22
ve_90	0.31 ± 0.16	0.18 ± 0.10	0.44 ± 0.21	<0.001
ve_skew	5.18 ± 2.32	6.19 ± 3.82	4.74 ± 2.20	0.092
ve_kurt	70.67 ± 48.16	85.37 ± 49.43	64.37 ± 34.92	0.068
kep_max	2.04 ± 0.84	2.54 ± 1.52	1.83 ± 0.91	0.012
kep_90	0.65 ± 0.24	0.80 ± 0.45	0.59 ± 0.30	0.016
kep_skew	4.05 ± 2.16	4.28 ± 2.37	3.95 ± 2.12	0.56
kep_kurt	68.18 ± 32.96	58.86 ± 28.09	72.17 ± 37.80	0.087

### PDZK1 is up-regulated in cancer tissues of HGG patients

**Fig. 1:**
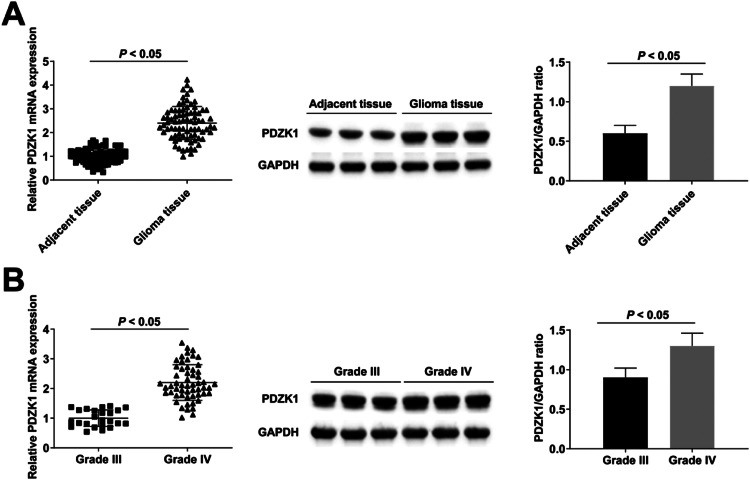
**Up-regulated expression of PDZK1 in cancer tissues of HGG patients.** (A‒B) RT-qPCR and Western blot detection of PDZK1 expression in HGG patients.

RT-qPCR and Western blot found that PDZK1 in cancer tissues was up-regulated compared with para-cancer tissues (Fig. 1A), and PDZK1 in grade IV HGG patients was higher than in grade III HGG patients (Fig. 1B).

### Correlation between PDZK1 expression and DCE-MRI perfusion parameters

PDZK1 expression was significantly positively correlated with K^trans^_90_, v_e_90_, and negatively correlated with k_ep_max_, k_ep_90_ (Table 2).

**Table 2:** Correlation between PDZK1 expression and DCE-MRI perfusion parameters.

	PDZK1	
Perfusion parameters	r	p
K^trans^_90_	0.432	0.006
v_e_90_	0.414	0.007
k_ep_max_	−0.338	0.034
k_ep_90_	−0.365	0.021

### Relationship between PDZK1 expression and prognosis of patients

**Fig. 2:**
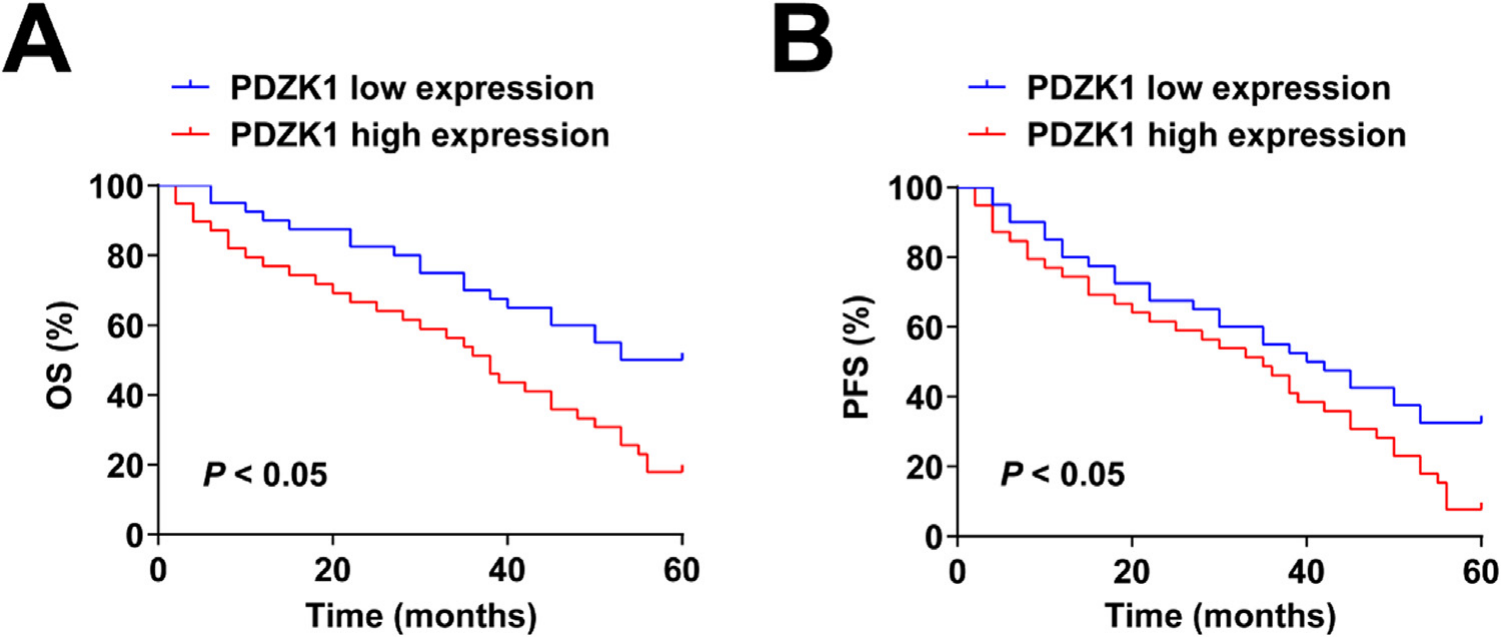
**Patients with high PDZK1 expression have poor OS and PFS.** (A‒B) Kaplan-Meier curve analysis and log-rank test results.

Cox regression analysis showed that age, glioma grade, v_e_90_, v_p_skew_, PDZK1, v_p_kurt_, and k_ep_90_, were significant predictors of OS, and age, Glioma grade, v_e_90_, v_e_skew_, and PDZK1 were predictive indicators for PFS (Table 3). Patients with high PDZK1 expression had poor OS and PFS (Fig. 2A‒B).

**Table 3:** Cox regression analysis evaluated the risk factors affecting survival.

Parameters	Overall survival			Progression-free survival		
	HR	95 % CI	p	HR	95 % CI	p
Age	1.08	1.04‒1.12	0.001	1.04	1.02‒1.07	<0.001
Glioma grade	7.01	1.57‒29.83	0.006	3.44	1.58‒7.45	0.003
v_p_skew_	1.16	1.01‒1.33	0.021	0.82	0.46‒1.44	0.472
v_p_kurt_	1.04	1.02‒1.06	0.007	0.87	0.50‒1.51	0.527
v_e_90_	3.15	1.28‒7.99	0.026	2.7	1.23‒5.95	0.015
v_e_skew_	0.92	0.85‒1.00	0.08	0.93	0.87‒0.99	0.036
k_ep_90_	0.45	0.20‒0.97	0.045	0.78	0.50‒1.20	0.249
PDZK1	2.12	1.16‒5.32	0.001	1.78	1.07‒4.81	<0.001

### Diagnostic value of PDZK1 expression

**Fig. 3:**
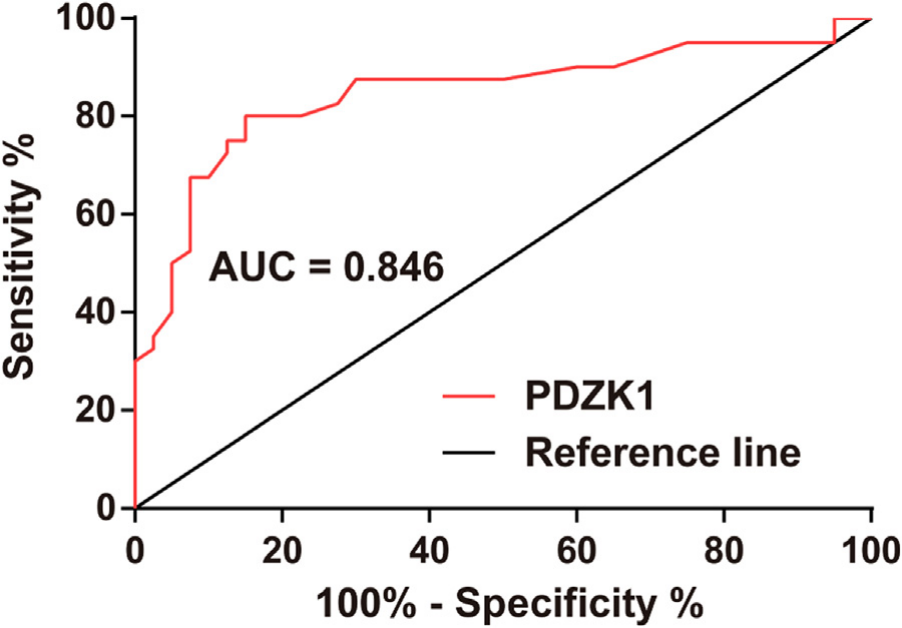
**PDZK1 expression can distinguish between grade III and grade IV gliomas.** ROC curve analysis of the diagnostic value of PDZK1 in glioma grading.

ROC curve analysis clarified that PDZK1 expression could distinguish between grade III and grade IV HGG (Fig. 3).

## Discussion

In malignant gliomas, neovascularization consisting of endothelial cells and structurally incomplete basement membranes is increased and its morphology is highly irregular, in which increased vascular resistance and pressure may lead to increased vascular permeability, rupture, and bleeding. To some extent, DCE-MRI reflected that the above pathophysiological changes in tumor neovascularity were somehow associated with glioma malignancy.^19^^,^^20^ As oligodendroglioma has different perfusion characteristics, DCE perfusion analysis may be biased,^21^ so astrocytomas were analyzed in this study. In this study, histogram analysis was used instead of the ROI maximization method, because histogram has better reproducibility^22^ and can calculate skewness and kurtosis in tumor voxels.

The lack of research on the other than K^trans^ parameters could be because only K^trans^ was originally recommended as the primary end point for perfusion studies.^23^ However, even the correlation between Ktrans and survival has not been firmly established as well. In an early study on the topic, Mills et al.^24^ surprisingly found higher K^trans^ to result in longer survival. This trend is indeed observed in some non-brain tumors: metastatic renal cell carcinoma and possibly hepatocellular carcinoma treated with vascular endothelial growth factor inhibitors,^25^ as well as head and neck squamous cell carcinoma treated with chemoradiation.^26^ Higher K^trans^ is associated with prolonged OS presumably by enhanced drug delivery through more permeable capillaries in the tumor. However, subsequent glioma studies have shown the opposite trend. Gliomas with lower vascular permeability (K^trans^) and a pronounced decrease of it on the course of treatment are associated with longer OS after chemotherapy^27^ and radiotherapy.^28^ v_e_ stands for extracellular volume fraction or leakage space. Contrast agents accumulate in the intravascular lumen after they escape,^29^ so v_e_ is considered to reflect tumor extravascular structure.^30^ In preclinical studies, v_e_ has been found to correspond well to the histological extracellular volume,^31^ coincide with histological necrosis and apoptosis,^32^ and correlate negatively with cellularity in glioma models.^33^ V_p_ represents the ratio of plasma volume to unit tissue volume.^34^ Tumor vascularization may be related to tumor aggressiveness.^35^ k_ep_ represents the reverse transfer of contrast agents from extravascular to intravascular areas. In the case of large v_e_, the transferred contrast agent is easy to accumulate here, resulting in retrograde transfer delay.^36^ In the present study, significant differences were recognized in some perfusion parameters between grade III and IV gliomas, with K_trans 90_, and v_e_90_ being the best differentiators. These two parameters differ between grade III and grade IV,^10^ high-grade and low-grade,^37^ and grade II and grade III oligodendroglioma.^38^ Meanwhile, k_ep_max_ and k_ep_90_ were lower in grade IV glioma.

PDZK1, a 70-kDa adapter protein with four PDZ-interacting domains. Some studies have suggested that PDZK1 is up-regulated in hepatocellular carcinoma^39^ and cervical cancer,^40^ but PDZK1 in glioma has not been studied. This study found that PDZK1 was upregulated in cancer tissues of HGG patients, and patients with high PDZK1 expression had poor OS and PFS. Moreover, PDZK1 expression can distinguish between grade III and grade IV gliomas. PDZK1 expression was significantly positively correlated with K^trans^_90_, and v_e_90_, and negatively correlated with k_ep_max_, and k_ep_90_.

This study has some limitations. First, the sample size was relatively small (*n* = 80), and the results of the study may be subject to some error. Second, it is challenging to assess the correlation between DCE-MRI abnormal signals and protein expression in glioma tissues. Therefore, a larger sample size and more appropriate methods should be used to test these results in future studies. In addition, the authors did not assess the diagnostic value of DCE perfusion parameters for glioma grading. However, the main purpose of this study was to investigate the expression of PDZK1 in HGG patients and its relationship with DCE perfusion parameters. The diagnostic value of DCE perfusion parameters for glioma grading awaits future studies.

## Conclusion

PDZK1 is up-regulated in HGG, and high expression of PDZK1 predicts poor PFS and OS in HGG patients. PDZK1 shows good diagnostic performance in differentiating grade III and grade IV gliomas and is correlated with DCE-MRI perfusion parameters.

## Ethical statement

All procedures performed in this study involving human participants were in accordance with the ethical standards of the institutional and/or national research committee and with the 1964 Helsinki Declaration and its later amendments or comparable ethical standards. All subjects were approved by The First People's Hospital of Shuangliu District (n° 201906SC29).

## Availability of data and materials

The data that support the findings of this study are available from the corresponding author, upon reasonable request.

## Authors' contributions

Conceptualization, Y. Zhang; methodology, Y. Zhang, F. Wang and Y.L. Huang; formal analysis, Y. Zhang; investigation, F. Wang and Y.L. Huang; data curation, F. Wang and Y.L. Huang; writing-original draft preparation, Y. Zhang; writing-review and editing, Y. Zhang and F. Wang; project administration, Y. Zhang. All authors have read and agreed to the published version of the manuscript.

## Funding

Not applicable.

## Conflicts of Interest

The authors declare no conflicts of interest.

## References

[1] Wen P., Kesari S. (2008). Malignant gliomas in adults. N Engl J Med.

[2] Smoll N., Schaller K., Gautschi O. (2013). Long-term survival of patients with glioblastoma multiforme (GBM). J Clin Neurosci.

[3] Theeler B., Yung W., Fuller G., De Groot J (2012). Moving toward molecular classification of diffuse gliomas in adults. Neurology.

[4] Ducray F., El Hallani S., Idbaih A (2009). Diagnostic and prognostic markers in gliomas. Curr Opin Oncol.

[5] Saraswathy S., Crawford F., Lamborn K., Pirzkall A., Chang S., Cha S. (2009). Evaluation of MR markers that predict survival in patients with newly diagnosed GBM prior to adjuvant therapy. J Neurooncol.

[6] Hirai T., Murakami R., Nakamura H., Kitajima M., Fukuoka H., Sasao A. (2008). Prognostic value of perfusion MR imaging of high-grade astrocytomas: long-term follow-up study. AJNR Am J Neuroradiol.

[7] Law M., Young R., Babb J., Peccerelli N., Chheang S., Gruber M. (2008). Gliomas: predicting time to progression or survival with cerebral blood volume measurements at dynamic susceptibility-weighted contrast-enhanced perfusion MR imaging. Radiology.

[8] Aronen H., Gazit I., Louis D., Buchbinder B., Pardo F., Weisskoff R. (1994). Cerebral blood volume maps of gliomas: comparison with tumor grade and histologic findings. Radiology.

[9] Russell S., Elliott R., Forshaw D., Golfinos J., Nelson P., Kelly P (2009). Glioma vascularity correlates with reduced patient survival and increased malignancy. Surg Neurol.

[10] Arevalo-Perez J., Peck K., Young R., Holodny A., Karimi S., Lyo J. (2015). Dynamic contrast-enhanced perfusion mri and diffusion-weighted imaging in grading of gliomas. J Neuroimaging.

[11] Jain K., Sahoo P., Tyagi R., Mehta A., Patir R., Vaishya S. (2015). Prospective glioma grading using single-dose dynamic contrast-enhanced perfusion MRI. Clin Radiol.

[12] Li H., Zhang B., Liu Y., Yin C. (2014). EBP50 inhibits the migration and invasion of human breast cancer cells via LIMK/cofilin and the PI3K/Akt/mTOR/MMP signaling pathway. Med Oncol.

[13] Kim H., Abd Elmageed Z., Davis C., El-Bahrawy A., Naura A., Ekaidi I. (2014). Correlation between PDZK1, Cdc37, Akt and breast cancer malignancy: the role of PDZK1 in cell growth through Akt stabilization by increasing and interacting with Cdc37. Mol Med.

[14] Wang N., Zheng Y., Zhang X., Xu S., Li X., Meng Y. (2022). Roles of circ_0000135/miR-140-3p/PDZK1 network in cervical cancer. Clin Transl Oncol.

[15] Wen P.Y., Macdonald D.R., Reardon D.A., Cloughesy T.F., Sorensen A.G., Galanis E. (2010). Updated response assessment criteria for high-grade gliomas: response assessment in neuro-oncology working group. J Clin Oncol.

[16] Tofts P.S., Kermode A.G. (1991). Measurement of the blood-brain barrier permeability and leakage space using dynamic MR imaging. 1. Fundamental concepts. Magn Reson Med.

[17] Orton M.R., d'Arcy J.A., Walker-Samuel S., Hawkes D.J., Atkinson D., Collins D.J. (2008). Computationally efficient vascular input function models for quantitative kinetic modelling using DCE-MRI. Phys Med Biol.

[18] Leach M.O., Morgan B., Tofts P.S., Buckley D.L., Huang W., Horsfield M.A. (2012). Imaging vascular function for early stage clinical trials using dynamic contrast-enhanced magnetic resonance imaging. Eur Radiol.

[19] Jia Z., Geng D., Xie T., Zhang J., Liu Y. (2012). Quantitative analysis of neovascular permeability in glioma by dynamic contrast-enhanced MR imaging. J Clin Neurosci.

[20] Park M., Kim H., Jahng G., Ryu C., Park S., Kim S. (2009). Semiquantitative assessment of intratumoral susceptibility signals using non-contrast-enhanced high-field high-resolution susceptibility-weighted imaging in patients with gliomas: comparison with MR perfusion imaging. AJNR Am J Neuroradiol.

[21] Mangla R., Ginat D., Kamalian S., Milano M., Korones D., Walter K. (2014). Correlation between progression free survival and dynamic susceptibility contrast MRI perfusion in WHO grade III glioma subtypes. J Neurooncology.

[22] Heye T., Merkle E., Reiner C., Davenport M., Horvath J., Feuerlein S. (2013). Reproducibility of dynamic contrast-enhanced MR imaging. Part II. Comparison of intra- and interobserver variability with manual region of interest placement versus semiautomatic lesion segmentation and histogram analysis. Radiology.

[23] Leach M.O., Brindle K.M., Evelhoch J.L., Griffiths J.R., Horsman M.R., Jackson A. (2005). The assessment of antiangiogenic and antivascular therapies in early-stage clinical trials using magnetic resonance imaging: issues and recommendations. Br J Cancer.

[24] Mills S.J., Patankar T.A., Haroon H.A., Baleriaux D., Swindell R., Jackson A. (2006). Do cerebral blood volume and contrast transfer coefficient predict prognosis in human glioma?. AJNR Am J Neuroradiol.

[25] O'Connor J.P., Jayson G.C. (2012). Do imaging biomarkers relate to outcome in patients treated with VEGF inhibitors?. Clin Cancer Res.

[26] Bernstein J.M., Homer J.J., West C.M. (2014). Dynamic contrast-enhanced magnetic resonance imaging biomarkers in head and neck cancer: potential to guide treatment? A systematic review. Oral Oncol.

[27] Kickingereder P., Wiestler B., Graf M., Heiland S., Schlemmer H., Wick W. (2015). Evaluation of dynamic contrast-enhanced MRI derived microvascular permeability in recurrent glioblastoma treated with bevacizumab. J Neurooncology.

[28] Bisdas S., Smrdel U., Bajrovic F., Surlan-Popovic K (2016). Assessment of progression-free-survival in glioblastomas by intratreatment dynamic contrast-enhanced MRI. Clin Neuroradiol.

[29] Tofts P., Brix G., Buckley D., Evelhoch J., Henderson E., Knopp M. (1999). Estimating kinetic parameters from dynamic contrast-enhanced T(1)-weighted MRI of a diffusable tracer: standardized quantities and symbols. J Magn Reson Imaging.

[30] Leach M., Brindle K., Evelhoch J., Griffiths J., Horsman M., Jackson A. (2005). The assessment of antiangiogenic and antivascular therapies in early-stage clinical trials using magnetic resonance imaging: issues and recommendations. Br J Cancer.

[31] Aref M., Chaudhari A., Bailey K., Aref S., Wiener E. (2008). Comparison of tumor histology to dynamic contrast enhanced magnetic resonance imaging-based physiological estimates. Magn Reson Imaging.

[32] Pike M., Stoops C., Langford C., Akella N., Nabors L., Gillespie G. (2009). High-resolution longitudinal assessment of flow and permeability in mouse glioma vasculature: sequential small molecule and SPIO dynamic contrast agent MRI. Magn Reson Med.

[33] Aryal M., Nagaraja T., Keenan K., Bagher-Ebadian H., Panda S., Brown S. (2014). Dynamic contrast enhanced MRI parameters and tumor cellularity in a rat model of cerebral glioma at 7 T. Magn Reson Med.

[34] Tofts P., Wicks D., Barker G. (1991). The MRI measurement of NMR and physiological parameters in tissue to study disease process. Prog Clin Biol Res.

[35] Nguyen T., Cron G., Mercier J., Foottit C., Torres C., Chakraborty S. (2015). Preoperative prognostic value of dynamic contrast-enhanced MRI-derived contrast transfer coefficient and plasma volume in patients with cerebral gliomas. AJNR Am J Neuroradiol.

[36] Yoo R.E., Choi S.H., Kim T.M., Park C.K., Park S.H., Won J.K. (2017). Dynamic contrast-enhanced MR imaging in predicting progression of enhancing lesions persisting after standard treatment in glioblastoma patients: a prospective study. Eur Radiol.

[37] Jia Z., Geng D., Liu Y., Chen X., Zhang J. (2013). Low-grade and anaplastic oligodendrogliomas: differences in tumour microvascular permeability evaluated with dynamic contrast-enhanced magnetic resonance imaging. J Clin Neurosci.

[38] Arevalo-Perez J., Kebede A., Peck K., Diamond E., Holodny A., Rosenblum M. (2016). Dynamic contrast-enhanced MRI in low-grade versus anaplastic oligodendrogliomas. J Neuroimaging.

[39] Guo L., Jiang W., Quan L., Teng X., Zhao J., Qiu H. (2022). Mechanism of PDZK1 in hepatocellular carcinoma complicated with hyperuricemia. J Oncol.

[40] Jiang Z., Cui X., Qu P., Shang C., Xiang M., Wang J. (2022). Roles and mechanisms of puerarin on cardiovascular disease: a review. Biomed Pharmacother.

